# Effects of red palm oil intervention on serum lipids, hepatic antioxidant capacity and gut microbiota in high-fat diet-fed mice

**DOI:** 10.1515/biol-2025-1350

**Published:** 2026-07-13

**Authors:** Chengyu Ma, Hairui Yu, Lingyao Li, Jiayi Zhang, Leyong Yu, Xiaojing Wu, Govindhrajan Sattanathan, Baobin Lu

**Affiliations:** College of Fisheries and Life Science, Shanghai Ocean University, Shanghai, 201306, China; Key Laboratory of Biochemistry and Molecular Biology in Universities of Shandong (Weifang University), Weifang Key Laboratory of Coho Salmon Culturing Facility Engineering, Institute of Modern Facility Fisheries, College of Advanced Agriculture and Life Sciences, Weifang University, Weifang, 261061, China; Shandong Freshwater Fisheries Research Institute, Jinan, 250013, China; Shandong Collaborative Innovation Center of Coho Salmon Health Culture Engineering Technology, Shandong Conqueren Marine Technology Co. Ltd., Weifang, 261108, China; Department of Physiology and Pharmacology, Karolinska Institutet, Stockholm, 17177, Sweden; College of Fisheries, Huazhong Agricultural University, Wuhan, 430070, China; Weifang Key Laboratory of Salmon and Trout Health Culture, Conqueren Leading Fresh Science and Technology Inc. Ltd., Weifang, 261205, China

**Keywords:** high fat diet, gut microbiota, serum lipid levels, hepatic antioxidant capacity, red palm oil

## Abstract

High-fat diets (HFDs) can induce oxidative stress and gut microbiota imbalance, highlighting the need to explore functional dietary lipids such as red palm oil (RPO) for metabolic health improvement. This study investigated the effects of RPO intervention administered through oral gavage on growth performance, serum lipid profile, hepatic antioxidant capacity, and gut microbiota in HFD-fed mice. Compared to the high-fat control (HO) group, RPO treatment significantly reduced triglycerides (TG) and total cholesterol (TC), and increased high-density lipoprotein cholesterol (HDL-C). Furthermore, hepatic antioxidant capacity was markedly enhanced, as evidenced by improved superoxide dismutase (SOD) and catalase (CAT) activities and decreased malondialdehyde (MDA) levels in the HFD group receiving RPO. Moreover, RPO supplementation reduced serum alanine aminotransferase (ALT) and aspartate aminotransferase (AST), and attenuated hepatic steatosis. Gut microbiota analysis showed that RPO administration enhanced the relative abundance of beneficial bacteria while reducing potentially harmful taxa. Overall, these findings demonstrate that oral gavage of RPO can effectively alleviate HFD-induced metabolic disturbances by improving lipid metabolism, enhancing hepatic antioxidant defenses, and modulating gut microbial composition.

## 1 Introduction

Modern dietary patterns characterized by high-fat intake have been closely associated with the growing global incidence of metabolic disorders, including non-alcoholic fatty liver disease (NAFLD), dyslipidemia, and obesity [1], with nutrition-related health challenges being reported across diverse settings worldwide [2]. Diets rich in fat have been shown to induce oxidative stress, chronic inflammation, and gut microbial dysbiosis, all of which play significant roles in the development and progression of metabolic diseases [3]. Different experimental models utilizing mice subjected to a high-fat diet (HFD) have been extensively applied to explore the mechanisms linking dietary fat intake to metabolic diseases, as well as to evaluate potential nutritional strategies for metabolic regulation [4]. Mice on an HFD exhibit remarkable metabolic alterations, such as increased body weight and adiposity, raised serum lipid levels, hepatic oxidative damage, and modifications in gut microbial composition, which closely resemble the characteristics of human metabolic syndrome [3].

Oxidative stress is a common pathological mechanism that can be triggered by various factors, including drugs, bacterial infections, intense exercise and HFDs [5], [6], [7], [8]. Emerging evidence underscores the major role of oxidative stress in metabolic disturbances induced by HFDs. A high intake of dietary fats contributes to elevated reactive oxygen species (ROS), particularly in the liver, a critical organ for lipid metabolism. The resultant elevated ROS levels lead to lipid peroxidation and disruption of metabolic pathways, thereby aggravating metabolic dysfunction. The liver has an endogenous antioxidant defense system that contains enzymes such as superoxide dismutase (SOD), catalase (CAT), and glutathione peroxidase, which are essential for neutralizing oxidative damage [9]. However, chronic ingestion of an HFD damages these antioxidant defenses. Furthermore, oxidative stress is closely associated with systemic inflammatory signaling, which contributes to the exacerbation of metabolic disturbances and liver pathology.

Given the increasing global interest in sustainable and functional dietary strategies for metabolic health, interventions that concurrently address lipid metabolism, oxidative stress, and gut microbial balance are of significant interest [10]. Among the dietary components with potential therapeutic value, functional lipids containing bioactive antioxidant compounds have garnered attention. Red palm oil (RPO) is particularly rich in carotenoids, tocopherols, and tocotrienols [11], [12], [13]. Lipid-soluble antioxidants demonstrate significant free radical scavenging activity and have been examined for their potential roles in the modulation of oxidative stress and lipid metabolism [14]. The distinct biochemical composition of RPO sets it apart from other vegetable oils and indicates its potential impact on metabolic health through various pathways [15]. Tocotrienols, which are abundant in RPO, have been extensively studied for their antioxidative, anti-inflammatory, and lipid-modulating properties [16]. In animal models, supplementation with tocotrienol-rich RPO has demonstrated efficacy in improving serum lipid profiles, reducing hepatic lipid accumulation, and enhancing antioxidant enzyme activities under conditions of diet-induced metabolic stress [17], 18]. Specifically, the inclusion of tocotrienols in the diet was found to mitigate hyperlipidemia and oxidative stress resulting from an HFD in mice, resulting in enhanced lipid homeostasis and decreased levels of inflammatory markers [19]. Short-term rat toxicity studies provide additional evidence for the biosafety of RPO at normal intake levels, showing no negative impacts on body weight, organ indices, or hematological and biochemical parameters [20]. These outcomes collectively characterize RPO as a safe edible oil rich in bioactive compounds, making it appropriate for the development of functional foods targeted at enhancing metabolic health. Furthermore, recent research indicates that the bioactive constituents of RPO may have the potential to influence gut microbiota, suggesting a significant mechanism through which nutritional antioxidants could exert metabolic effects in the host [21].

Despite mounting evidence supporting the metabolic benefits of RPO, there is a scarcity of research investigating its comprehensive impacts on serum lipid metabolism, hepatic antioxidant capacity, and gut microbiota composition. Therefore, this study utilized a murine HFD to evaluate the effects of RPO intervention on growth performance, serum lipid levels, hepatic antioxidant capacity, liver injury markers, and gut microbiota composition. The objective of this investigation is to propose a natural dietary intervention aimed at protecting metabolic health in the context of high-fat dietary patterns.

## 2 Materials and methods

### 2.1 Reagents and animals

The RPO was acquired from Dama Palm Oil Technology R&D (Shanghai) Co., Ltd., located in Shanghai, China. The olive oil was obtained from Shandong Luhua Group Co., Ltd., based in Yantai, China. The high-fat feed, contributing 60 % of energy from fat, and the low-fat feed, contributing 10 % of energy from fat, were sourced from Shuyu Biotechnology Co., Ltd. in Shanghai, China. Diet formulations are shown in Table 1.

**Table 1:** Experimental diet composition shown as g/100 g.

Ingredients	Low-fat diet	High-fat diet
Casein	25.85	18.96
L-cystine	0.39	0.28
Corn starch	0.00	47.98
Maltodextrin	16.15	11.85
Sucrose	9.41	6.90
Cellulose	6.46	4.74
Soybean oil	3.23	2.37
Lard	31.66	1.90
Mineral mix	6.46	4.74
Vitamin mix	0.13	0.10
Choline bitartrate	0.26	0.19
Total	100.00	100.00
Kcal/100 g	381.00	520.00
Protein	20.00 %	20.00 %
Fat	10.00 %	60.00 %
Carbohydrate	70.00 %	20.00 %

The study utilized thirty-two 8-week-old male mice (C57BL/6J) obtained from Shandong Emodacom Life Science Co., Ltd, [No. SCXK (Lu) 20230010, Weifang, China]. The mice were maintained at a constant temperature of 23 ± 3 °C and a humidity of 50 ± 5 %. The environment was kept clean, quiet, and well-ventilated, with *ad libitum* access to a low-fat diet and water.

**Ethical approval:** The research related to animal use has been complied with all the relevant national regulations and institutional policies for the care and use of animals, and has been approved by the Animal Ethical and Welfare Committee of the Institute of Modern Facility Fisheries (NO. 20240316).

### 2.2 Experiment designs and feeding protocols

After one week of acclimatization, 32 mice were randomly allocated into four groups according to their body weight, with an overall mean initial body weight of 19 ± 2 g. Mice in the low-fat control group (LO) received a low-fat diet; whereas those in the high-fat control (HO), high-fat low-dose RPO (RLO), and high-fat high-dose RPO (RHO) groups were fed an HFD for 10 weeks. The LO and HO groups received olive oil by oral gavage at a dose of 10 mL/kg/day; the RLO group received 10 mL/kg/day of a mixed oil preparation (RPO: olive oil = 3:7), and the RHO group received 10 mL kg/day of RPO. The gavage volume of 10 mL/kg/day was selected according to the OECD guidelines for non-aqueous vehicles in rodents and with reference to a 28-day short-term toxicity study of red palm oil [20], 22]. For all groups, the administered volume corresponded to approximately 1 % of each mouse’s body weight, and the gavage volume was kept identical across groups to eliminate potential confounding effects associated with the gavage procedure itself. During the experimental period, mice were housed in standard rearing cages with *ad libitum* access to food and water. Daily food intake was measured at the cage level, with body weight recorded daily.

### 2.3 Sampling procedures

Following the end of the experimental trial, mice underwent a 24-h fasting period prior to euthanasia, which was performed through an intraperitoneal injection of sodium pentobarbital (2 %, 40 mg/kg body weight). Blood was collected via the retro-orbital plexus and processed to obtain serum by centrifugation at 4 °C (3,200×*g*, 10 min). The serum supernatant was promptly stored at −80 °C for future analysis. After blood collection, liver samples were excised, rinsed with physiological saline, blotted dry using filter paper. Subsequently, portions of the liver tissues were stored at −80 °C for subsequent experimentation, while the remaining portions were fixed in 4 % paraformaldehyde solution for further processing. Cecal feces were aseptically collected and stored at −80 °C for 16S rRNA sequencing.

### 2.4 Serum biochemical analyses

Concentrations of serum lipids, including TG, total cholesterol (TC), HDL-C, and LDL-C, as well as the liver injury markers aspartate aminotransferase (AST) and alanine aminotransferase (ALT), were determined using enzymatic colorimetric methods. Measurements were performed using a Tecan Spark 10 M microplate reader (Tecan Group Ltd., Männedorf, Switzerland) with commercial assay kits obtained from Nanjing Jiancheng Bioengineering Institute (Nanjing, China), in strict accordance with the manufacturer’s protocols.

### 2.5 Liver biochemical analyses

The liver samples were processed into 10 % homogenates at 4 °C with a hand-held homogenizer following kit protocols, followed by centrifugation (3,200×*g*, 10 min). The supernatant was then collected for the determination of enzyme activities. The activities of superoxide dismutase (SOD) and catalase (CAT), as well as the contents of glutathione (GSH) and malondialdehyde (MDA) in the liver, were determined, were determined according to the manufacturer’s protocols with commercial kits from Nanjing Jiancheng Bioengineering Institute (Nanjing, China).

### 2.6 Liver histopathological analysis

Liver tissues were fixed in 4 % paraformaldehyde (Biosharp, Beijing, China) at room temperature for 24 h, dehydrated through an ascending ethanol gradient using an automated tissue processor (Servicebio, China) and embedded in paraffin with an embedding machine (Servicebio, China). Sections were cut at a thickness of 5 μm using the rotary microtome (Servicebio, China). After dewaxing in xylene and rehydration through a descending ethanol gradient to distilled water, the sections were stained with hematoxylin and eosin (H&E), dehydrated, cleared in xylene, and mounted with neutral balsam. Histopathological changes were examined and imaged under a light microscope (Nikon Co., Ltd., Tokyo, Japan) at 200× magnification. Liver histology was evaluated using the NAFLD Activity Score (NAS) by an experienced pathologist blinded to the experimental groups.

### 2.7 Gut microbiota analysis

Fecal genomic DNA was isolated with the TGuide S96 Kit (Tiangen Biotech Co., Ltd., Beijing, China). PCR amplification of the bacterial 16S rRNA gene V3–V4 region was carried out using the primers 338F (5′-ACT​CCT​ACG​GGA​GGC​AGC​A-3′) and 806R (5′-GGACTACHVGGGTWTCTAAT-3′) with the following thermal cycling conditions: 95 °C for 5 min; 25 cycles of 95 °C for 30 s, 50 °C for 30 s, 72 °C for 40 s; and 72 °C for 7 min. Paired-end sequencing was performed on the Illumina NovaSeq 6000 platform. The raw sequencing reads underwent initial quality control through Trimmomatic (v0.33), followed by primer trimming utilizing Cutadapt (v1.8.3). The processed sequences were then analyzed using the DADA2 plugin within the QIIME2 (v2020.6) pipeline for denoising, paired-end merging, and chimera removal. ASVs were defined at 100 % sequence identity, and taxonomic assignment was performed against the SILVA 138 database with a confidence threshold of 0.7. Data analysis was primarily conducted on the Qingke Biotechnology Cloud Platform (https://www.tsingke.com.cn), where alpha and beta diversity indices, along with differential species analysis (LEfSe), were computed using the QIIME2 (v2020.6) pipeline.

### 2.8 Statistical analyses

All data were presented as the mean ± standard deviation (SD). Group differences were assessed using one-way analysis of variance (ANOVA), followed by the Waller-Duncan test, a Bayesian multiple comparison procedure suitable for experiments with a modest number of treatment groups. For microbiota differential abundance and Spearman correlation analyses, *p*-values were adjusted using the Benjamini–Hochberg false discovery rate (BH-FDR) method, with adjusted *pvalue* <0.05 considered statistically significant. All statistical analyses were conducted using SPSS version 27.0. Graphs were generated using GraphPad Prism version 8.0.2.

## 3 Results

### 3.1 Body weight change and energy intake

**Figure 1:**
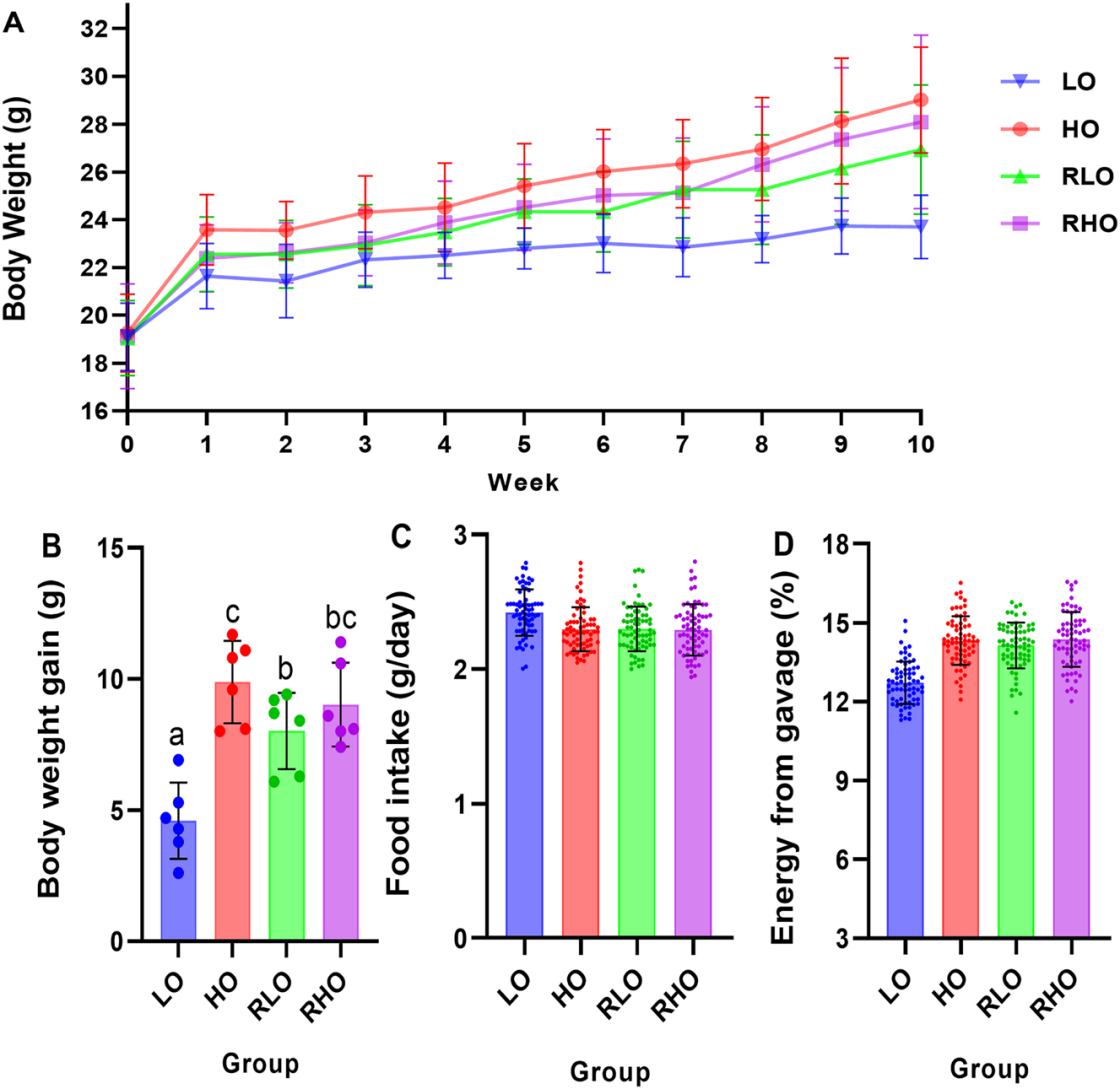
Body weight and energy intake parameters. (A) Body weight change curve. (B) Total body weight gain at the end of the experiment (n = 6, mean ± SD). Values with a different letter indicates significant at (*p* < 0.05). (C) Daily food intake per mouse, normalized from cage-level consumption. Individual daily data are overlaid. (D) Caloric contribution of oil gavage to total daily energy intake. RPO alone accounted for 4.32 % of daily energy intake in RLO and 14.65 % in RHO.

A significant time × group interaction was observed for body weight over the 10-week experimental period. Post hoc comparisons showed that body weight in the HO group was significantly higher than that in the LO group only from week 5 to week 10 (*p* < 0.05, Figure 1A). Figure 1B shows the analysis of final body weight gain. The HO group gained significantly more weight than the LO group (*p* < 0.05). Compared with the HO group, the RLO group gained significantly less (*p* < 0.05), whereas no significant difference was observed between the HO and RHO groups (*p* > 0.05). Throughout the experimental period, the average daily food intake per mouse was similar across all groups (Figure 1C). The proportion of total daily energy intake contributed by the gavaged lipids was comparable among all groups, ranging from approximately 12.7 % to 14.4 % (Figure 1D). RPO alone accounted for 4.24 % of daily energy intake in the RLO group and 14.38 % in the RHO group.

### 3.2 Serum lipid profiles

As shown in Table 2, the results indicate that compared to LO group, the HO group showed significantly increased levels of TG and TC, along with significantly decreased HDL-C (*p* < 0.05). After RPO intervention, both RLO and RHO significantly reduced TG and increased HDL-C in the HO group (*p* < 0.05), with RLO showing a more pronounced effect on HDL-C elevation. However, only RLO significantly reduced TC compared with the HO group (*p* < 0.05), while both RLO and RHO exhibited no significant effect on LDL-C (*p* > 0.05).

**Table 2:** Serum parameters related to lipid metabolism.

Parameter	LO	HO	RLO	RHO
TG (mmol/L)	3.61 ± 0.25^c^	5.08 ± 0.35^a^	3.96 ± 0.48^bc^	4.14 ± 0.19^b^
TC (mmol/L)	1.00 ± 0.08^c^	1.56 ± 0.14^a^	1.26 ± 0.13^b^	1.58 ± 0.25^a^
LDL-C (mmol/L)	1.14 ± 0.12^b^	1.48 ± 0.27^a^	1.36 ± 0.14^ab^	1.43 ± 0.21^ab^
HDL-C (mmol/L)	3.06 ± 0.14^a^	2.60 ± 0.18^c^	3.03 ± 0.15^a^	2.81 ± 0.16^b^

Data in the table are mean ± SD (*n* = 6). Different lowercase letters (a, b, c) in each row indicate significant differences among groups (*p* < 0.05).

### 3.3 Serum hepatic enzymes

**Figure 2:**
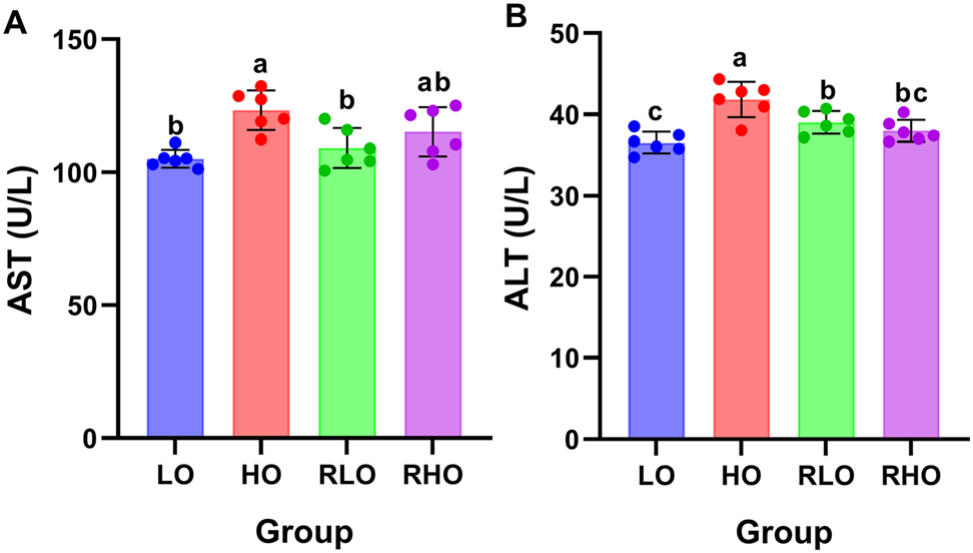
Impact of RPO on serum AST and ALT activities in mice. (A) AST activity; (B) ALT activity. Values are means ± SD, and the values with a different letter indicates significant at (*p* < 0.05).

The levels of AST and ALT were significantly higher in the HO group than the LO group (*p* < 0.05, Figure 2). AST and ALT levels in the RLO group were significantly lower than those in the HO group (*p* < 0.05). In the RHO group, AST levels fell between those of the LO and HO groups and did not differ significantly from either group (*p* > 0.05), while ALT levels were significantly lower than those in the HO group (*p* < 0.05).

### 3.4 Hepatic antioxidant capacity

**Figure 3:**
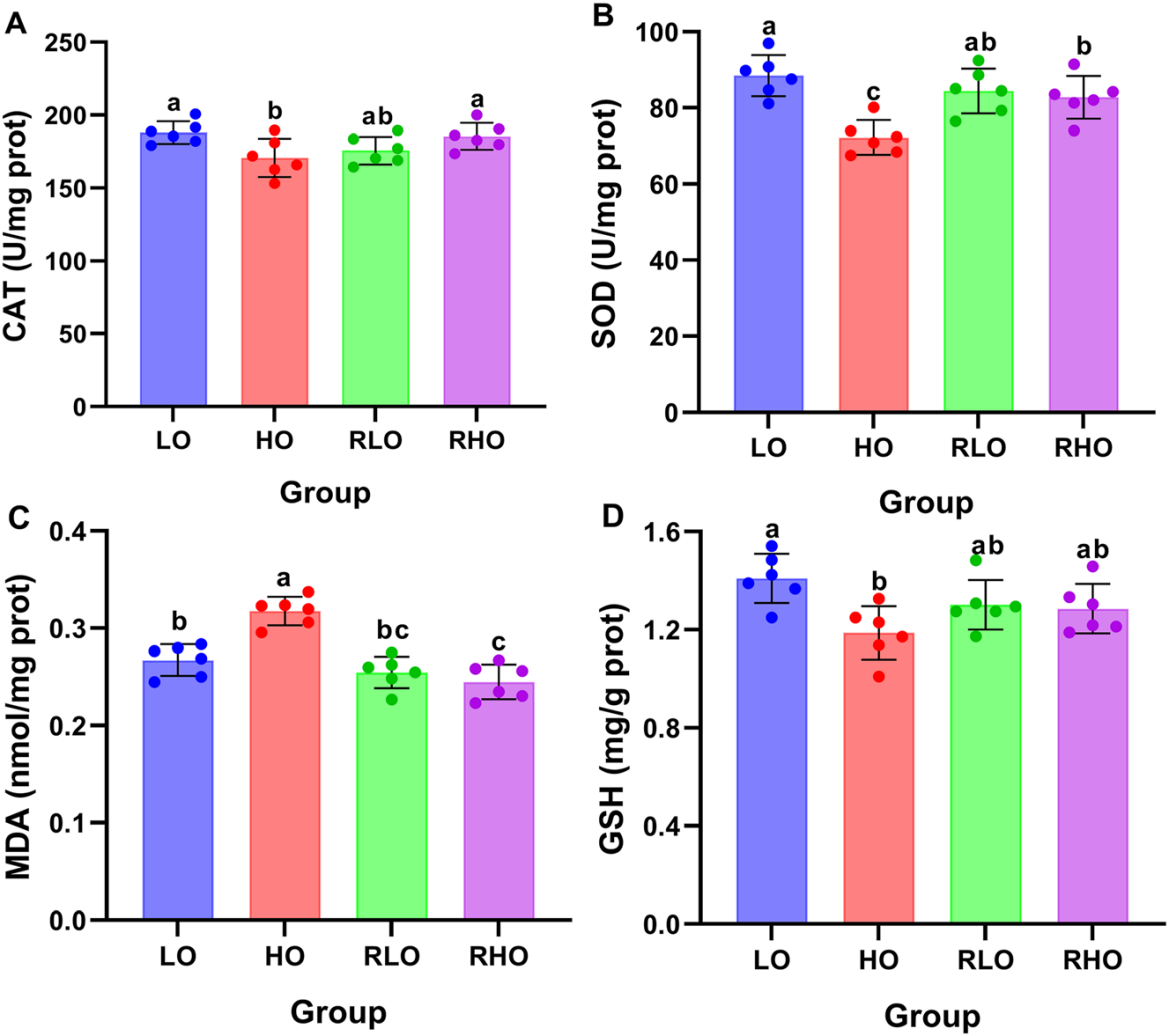
Impacts of RPO on antioxidant capacity in liver of mice. (A) CAT activities; (B) SOD activities; (C) MDA content; (D) GSH content. Values are means ± SD, and the values with a different letter indicates significant at (*p* < 0.05).

The dosage of RPO administered significantly affected the antioxidant capacity and MDA levels in the liver of the HFD-induced mice (Figure 3). Compared with the LO group, the HO group showed notably decreased the content of GSH and activities of SOD and CAT (*p* < 0.05). The RHO group showed a marked increase in CAT activity (*p* < 0.05). SOD activities were notably higher in both the RLO and RHO groups than in the HO (*p* < 0.05). Moreover, hepatic MDA levels were markedly lower in the LO, RLO and RHO groups than in the HO group (*p* < 0.05). No significant differences in the GSH contents were found among the HO, RLO, and RHO groups (*p* > 0.05); furthermore, neither the RLO nor the RHO groups differed notably from the LO group (*p* > 0.05). It is noteworthy that the HO group consistently showed the lowest GSH content.

### 3.5 RPO ameliorates hepatic steatosis in high-fat-fed mice

**Figure 4:**
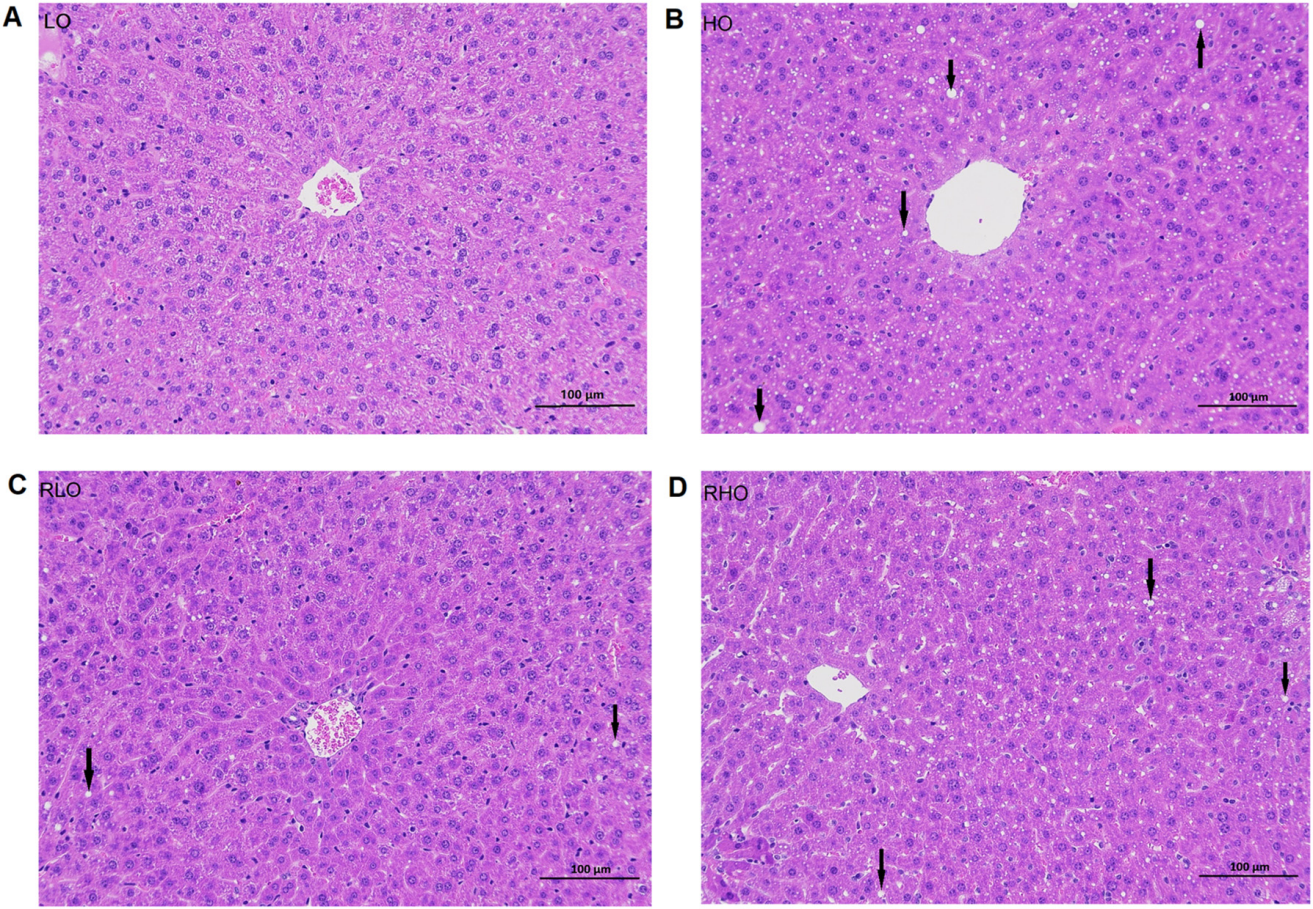
RPO ameliorates HFD-induced hepatic steatosis in mice. Representative H&E-stained liver sections. (A) LO group; (B) HO group; (C) RLO group; (D) RHO group. Magnification: 200×, scale bar = 100 μm. Black arrows indicate lipid vacuoles.

H&E staining of liver sections revealed varying degrees of hepatic steatosis across the experimental groups (Figure 4). In the LO group, hepatocytes exhibited normal morphology with almost no lipid vacuoles observed. In contrast, the HO group showed evident hepatic steatosis with observable lipid vacuoles in hepatocytes. The RHO group displayed a similar degree of steatosis to the HO group, with only a slight reduction in the number and size of lipid vacuoles. Meanwhile, in the RLO group only a minimal number of scattered lipid vacuoles were present, which was notably less severe than that in both the HO and RHO groups, though not completely absent. These observations were further confirmed by NAS scoring performed by a blinded pathologist.

#### 3.5.1 Alpha and beta diversity analysis

**Figure 5:**
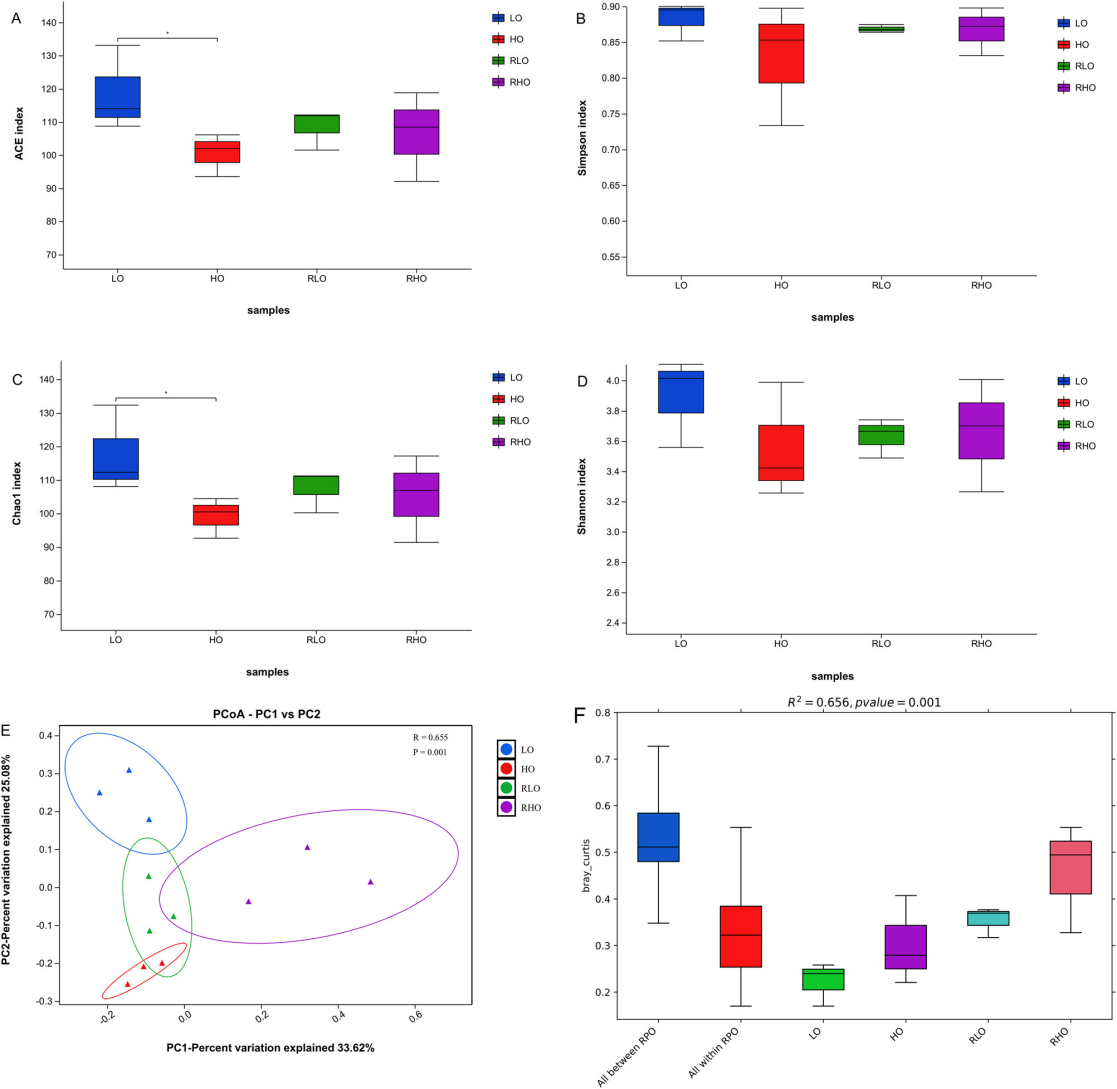
Analysis of gut microbiota composition, diversity, and associated biomarkers in mice from different groups. (A–D) Alpha diversity indices showing changes in species richness and evenness: (A) ACE index; (B) Simpson index; (C) Chao1 index; (D) Shannon index. (E) PCoA plot illustrated the distribution of gut microbial community structures among groups, with statistical tests for group differences. (F) Principal coordinate analysis (PCoA) based on Bray–Curtis distance. Group separation was tested by PERMANOVA (R^2^ = 0.656, *p* = 0.001).

Compared with the LO group, the HO group showed significantly reduced ACE and Chao1 richness indices (*p* < 0.05), while the Shannon and Simpson indices exhibited a non-significant decreasing trend (Figure 5A–D). No other significant differences were observed among the groups. β-diversity illustrates compositional variations among microbial communities. This investigation utilized principal coordinates analysis (PCoA) to systematically assess structural differences in gut microbiota among the experimental groups. As presented in Figure 5E, the LO group showed a distinct separation from the HO, RLO, and RHO groups along the PCo2 axis, with minimal overlap detected. Reproducibility within the groups was high, while the differences between groups were substantial. PERMANOVA confirmed significant differences in microbial community structure among groups (R^2^ = 0.656, *p* = 0.001, Figure 5F).

#### 3.5.2 Gut microbiota phylum-level and genus-level species composition

**Figure 6:**
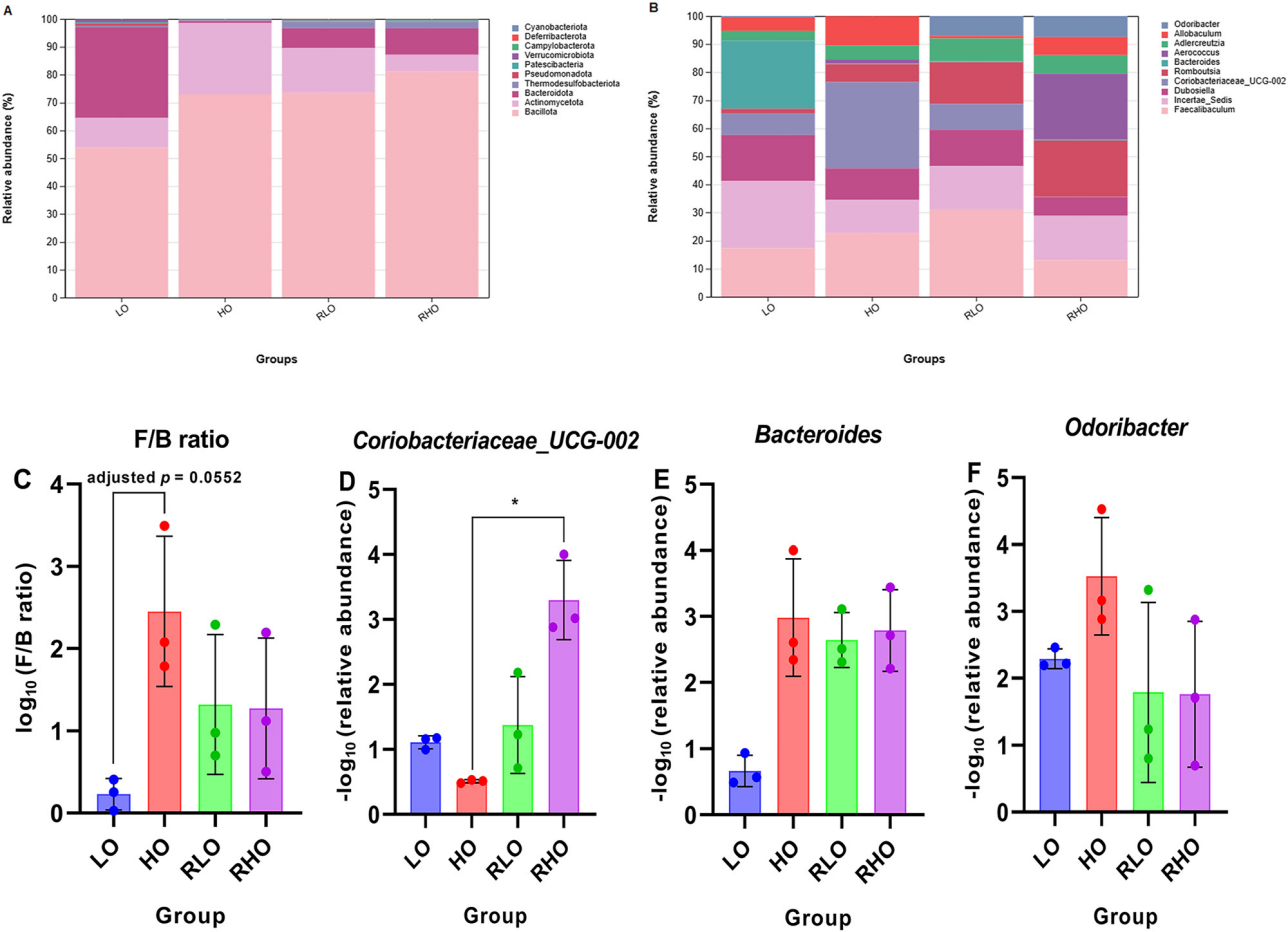
Gut microbiota composition. (A) Gut microbiota relative abundance at the phylum level. Bar plot shows the top 10 bacterial phyla averaged across all samples. (B) Gut microbiota relative abundance at the genus level. Bar plot shows the top 10 bacterial genera averaged across all samples; unclassified or low-abundance genera are grouped as “Incertae_Sedis”. (C–F) Histograms of the log10-transformed relative abundances of Firmicutes/Bacteroidota (F/B) ratio, Coriobacteriaceae_UCG-002, Bacteroides, and Odoribacter, respectively (n = 3, mean ± SD). Zero values were assigned a small pseudocount (0.0001) prior to log10 or −log10 transformation. Statistical comparisons were performed using Kruskal-Wallis test with Dunn’s post hoc test and BH-FDR correction; *p < 0.05.

At the phylum level (Figure 6A), the gut microbiota composition in all experimental groups was dominated by Bacteroidota (formerly Bacteroidetes) and Bacillota (formerly Firmicutes), whose combined relative abundance exceeded 70 %. As shown in Figure 6C, in comparison to the LO group, Bacillota tended to be higher in the HO group, while Bacteroidota tended to decrease, resulting in an increased F/B (Firmicutes/Bacteroidota) ratio (*p* > 0.05). At the genus level (Figure 6B, D-E), the overall stacked bar plot shows compositional differences among groups. *Coriobacteriaceae_UCG-002* was significantly higher in the HO group compared with RHO (*p* < 0.05, BH-FDR corrected). *Bacteroides* and *Odoribacter* did not reach statistical significance (*p* > 0.05). In the RLO and RHO groups, *Romboutsia*, *Adlercreutzia*, and *Odoribacter* showed numerical increases, and *Aerococcus* was relatively higher in the RHO group.

#### 3.5.3 LEfSe analysis and correlation analysis

**Figure 7:**
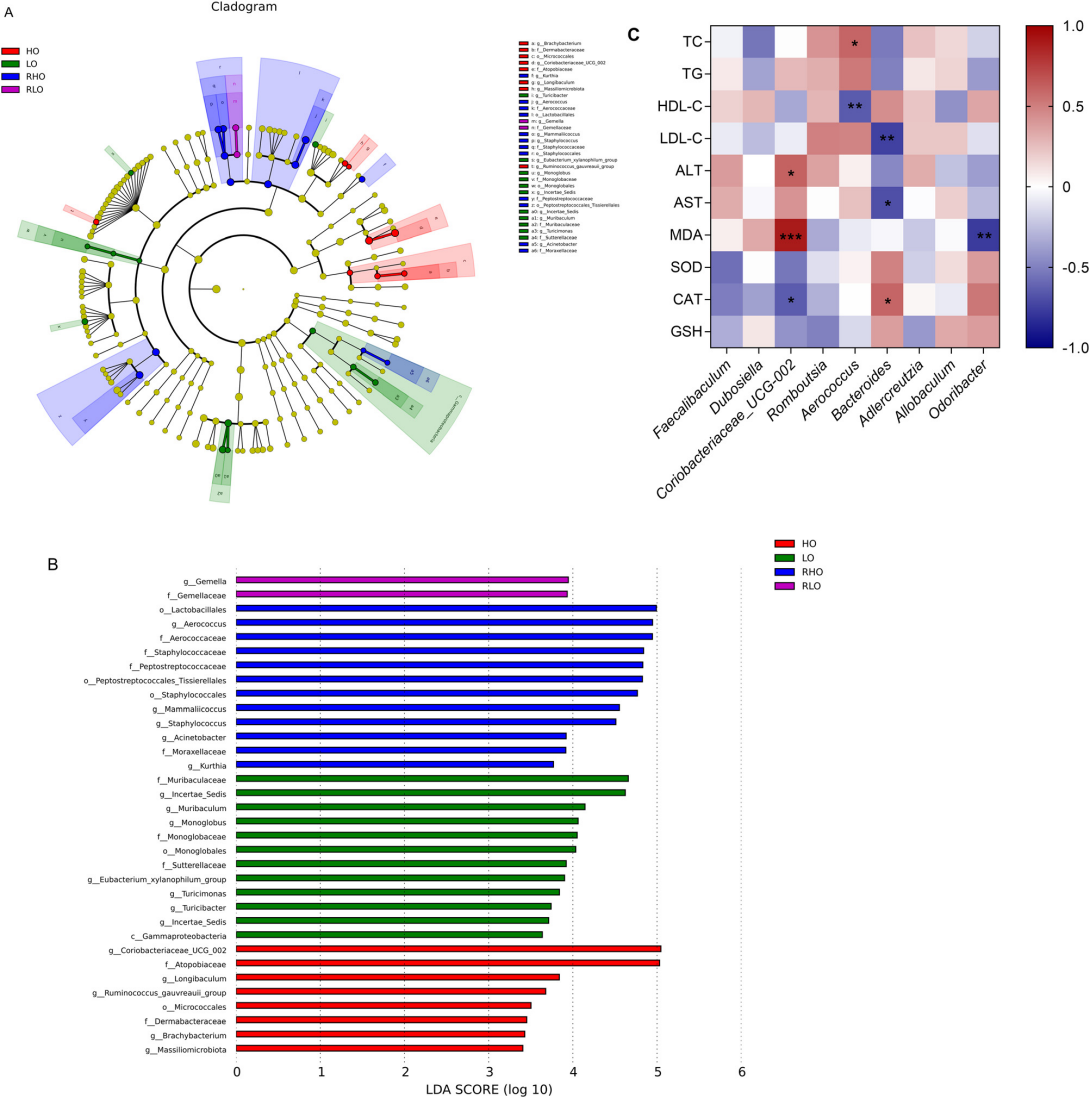
Identification of gut microbiota biomarkers and their associations with host phenotypes. (A) Hierarchical Cladogram generated by LEfSe analysis, illustrating the phylogenetic distribution of taxonomically differential and abundant taxa. (B) Histogram of LDA scores for the biomarker taxa identified by LEfSe analysis. (C) Spearman correlation heatmap between biomarker genera and host parameters. Blue indicates positive correlations, red indicates negative correlations, and white indicates near-zero correlations. ***Adjust *p* < 0.05 (FDR); ** *p* < 0.01 before correction; *p < 0.05 before correction.

LEfSe analysis identified microbial taxa with significant differential abundance across experimental groups. As shown in Figure 7A and B, taxa with LDA scores greater than 4 were primarily enriched in the HO and RHO groups. In the HO group, *Coriobacteriaceae_UCG-002* and its parent family Atopobiaceae were notably enriched (LDA > 5). In the RHO group, several taxa including *Aerococcus*, f_Aerococcaceae, o_Lactobacillales, f_Staphylococcaceae, and f_Peptostreptococcaceae exhibited LDA scores above 4, indicating strong group-specific enrichment. The LO group served as a baseline control for comparison and contained relatively fewer high-LDA taxa, primarily Muribaculaceae and its associated genera.

Spearman correlation analysis with BH-FDR correction revealed that *Coriobacteriaceae_UCG-002* was positively correlated with MDA (adjusted *p* < 0.05, Figure 7C). In addition, before FDR correction, *Bacteroides* showed positive correlations with CAT activity (*p* < 0.05) and AST levels (*p* < 0.01), together with a negative correlation with LDL-C levels (*p* < 0.05). *Aerococcus* was positively correlated with TC levels (*p* < 0.05) and negatively correlated with HDL-C levels (*p* < 0.01). *Odoribacter* abundance was negatively correlated with MDA levels (*p* < 0.01). Furthermore, *Coriobacteriaceae_UCG-002* showed a negative correlation with CAT activity (*p* < 0.05) and a positive correlation with ALT levels (*p* < 0.05). However, these associations did not remain statistically significant after BH-FDR correction.

## 4 Discussion

HFDs have been shown to disrupt energy metabolism and excessive lipid accumulation, leading to metabolic disorders such as obesity and insulin resistance [23], 24]. In the current study, mice fed an HFD exhibited higher body weight gain than those receiving a low-fat diet; however, low-dose RPO supplementation appeared to alleviate excessive body weight gain induced by HFD feeding. The positive impact of RPO on growth parameters may be linked to its distinctive composition of phytochemicals, including carotenoids, tocopherols, and tocotrienols [11]. Earlier investigation has shown that tocotrienols extracted from palm oil can enhance metabolic outcomes in animal models of HFD. For instance, supplementation with γ-tocotrienol in mice subjected to an HFD was shown to delay the onset of obesity and enhance insulin sensitivity by mitigating inflammation in adipose tissues [25]. Likewise, tocotrienol-rich fractions derived from palm oil were confirmed to modulate metabolic pathways associated with fatty acid oxidation and inflammation, thereby contributing to improved metabolic health in models of HFD-induced obesity [26].

Dyslipidemia is a common metabolic disorder associated with HFD intake. Elevated serum levels of TC and TG, along with reduced HDL-C levels, are typically observed in animals fed an HFD [27], 28], which is consistent with previous studies. The lipid-lowering effects observed in the RPO groups may be linked to its rich phytochemical profile. These bioactive compounds can inhibit hepatic cholesterol synthesis by suppressing HMG-CoA reductase activity, enhance lipoprotein lipase activity, and promote fatty acid β-oxidation pathways in hepatic and adipose tissues [11], 29]. Whether different doses of RPO differentially regulate these key lipid metabolic enzymes warrants further investigation, which may partly explain the different responses observed between the RLO and RHO groups. This non-linear pattern may reflect the complex nutritional composition of RPO, where the beneficial antioxidant effects of tocotrienols and carotenoids are partially offset by its saturated fatty acid content at higher doses. Future transcriptomic and proteomic studies are required to clarify the dose-dependent mechanisms. However, the specific contributions of individual tocotrienol isomers (α-, β-, γ-, and δ-forms) were not evaluated in this study and remain to be clarified in future work. The present findings suggest that RPO, which is rich in natural antioxidants, may exert beneficial effects on lipid metabolism when administered in controlled doses. However, the clinical significance of these lipid improvements for cardiovascular health remains to be further validated. Future studies should examine additional biomarkers such as inflammatory cytokines and endothelial function.

Oxidative stress and subsequent lipid peroxidation are critical mechanisms underlying HFD-induced liver injury [15]. In this study, HFD disrupted the hepatic redox balance, potentially compromising endogenous antioxidant defenses and initiating a cycle of oxidative damage. Supplementation with RPO effectively counteracted this process. Specifically, RPO significantly enhanced the activity of antioxidant enzymes (SOD and CAT), elevated hepatic GSH content, and reduced levels of the lipid peroxidation marker MDA. This restoration of antioxidant capacity aligns with prior studies demonstrating the antioxidant and cytoprotective effects of bioactive compounds in improving hepatic redox status across diverse experimental models [30], [31], [32], [33]. These observations support the current study’s conclusions, suggesting that RPO may confer protective effects on hepatic tissues against oxidative damage resulting from excessive dietary fat intake. The hepatoprotective and antioxidant properties of RPO may be associated with activation of the Nrf2/ARE signaling pathway, which has been reported to enhance the expression of endogenous antioxidant enzymes, including CAT and SOD [15]. The enhanced antioxidant responses observed following RPO supplementation suggest that different doses of RPO may exert distinct modulatory effects on Nrf2-related antioxidant defense mechanisms. Nevertheless, further transcriptomic and proteomic investigations are required to verify the regulatory roles of Nrf2 and its downstream antioxidant targets under conditions of HFD-induced oxidative stress.

Serum AST and ALT serve as widely recognized biomarkers for liver injury [34]. Elevated concentrations of these enzymes are typically indicative of hepatocellular damage and are regularly observed in animals exposed to an HFD, as a consequence of lipid accumulation and oxidative stress within liver tissues. The reduction in these enzymes after RPO treatment can be interpreted as a downstream functional benefit of the enhanced antioxidant defense. In the present study, RPO intervention markedly improved hepatic enzyme profiles in HFD-fed mice, with the RLO group showing the most pronounced effects, while the RHO group also demonstrated significant improvement relative to the HO group, further supporting the hepatoprotective capacity of RPO. This finding is supported by work showing that RPO’s bioactive constituents can promote hepatocyte stability under metabolic stress [35]. Thus, the coordinated improvements in both oxidative parameters and injury markers provide compelling evidence that RPO preserves hepatic structural and functional integrity primarily by mitigating oxidative stress.

Histological assessment of liver tissues offers valuable insights into the structural changes linked with metabolic disorders induced by HFDs. In this study, liver sections from mice subjected to an HFD showed significant histopathological alterations characterized by hepatic steatosis and lipid droplet accumulation, which is consistent with previous reports demonstrating that long-term high-fat feeding can induce NAFLD in experimental animals [36]. Conversely, mice treated with RPO showed significant improvements in liver histoarchitecture when compared to the HO group. Liver sections from RPO-treated mice demonstrated decreased lipid accumulation and enhanced hepatocyte morphology, suggesting a protective effect of RPO against hepatic damage induced by an HFD. These histological results correspond with the observed reductions in serum AST and ALT levels, indicating that RPO supplementation effectively mitigates hepatocellular injury.

In addition to the direct metabolic and histopathological effects observed, the investigation extended to the gut microbiota as a potential mechanistic pathway. Increasing evidence suggests that HFD-induced metabolic disorders are closely associated with gut microbial dysbiosis and oxidative stress through the gut–liver axis [37]. In the present study, HFD feeding tended to increase the F/B ratio and promoted the enrichment of *Coriobacteriaceae_UCG-002*, accompanied by elevated oxidative stress markers, suggesting that disruption of microbial homeostasis may contribute to HFD-associated metabolic impairment [38], [39], [40]. RPO supplementation partially reversed these microbial alterations and was accompanied by improvements in hepatic oxidative status and lipid metabolism. The F/B ratio has been widely recognized to have certain limitations as a standalone functional marker. In particular, RPO-treated groups exhibited increased abundances of genera such as *Odoribacter*, *Romboutsia*, and *Adlercreutzia*. Among these, *Odoribacter* is recognized as a short-chain fatty acid (SCFA)-producing bacterium involved in maintaining intestinal and metabolic homeostasis [41]. *Romboutsia* [42], a short-chain fatty acid (SCFA)-producing bacterium, and *Adlercreutzia* [43], which has been linked to the metabolism of dietary polyphenols and production of bioactive metabolites with antioxidant properties, also showed numerical increases in the RPO-treated groups. These metabolites have been suggested to be associated with activation of the Nrf2 antioxidant defense pathway and improved cellular resistance to oxidative stress. Their enrichment has been associated with improved metabolic outcomes in previous dietary intervention studies. SCFAs have been shown to participate in maintaining intestinal barrier integrity, regulating inflammatory responses, and modulating host energy metabolism [44], and have been demonstrated to activate AMPK and promote fatty acid β-oxidation. Therefore, the enrichment trend of *Odoribacter* following RPO supplementation may reflect an improvement in the intestinal microenvironment and may partially contribute to the enhanced antioxidant capacity and metabolic improvements observed in the present work. *Dubosiella*, which has been reported to alleviate HFD-induced dyslipidemia in mice [45], was also detected in the RPO-treated groups. In contrast, *Coriobacteriaceae_UCG-002* remained significantly enriched in the HO group and showed a positive correlation with hepatic MDA levels after BH-FDR correction, indicating a potential association between this taxon and oxidative stress under HFD conditions. Previous studies have linked members of the Coriobacteriaceae family with lipid metabolic disorders and inflammatory responses [46], further supporting its possible involvement in HFD-induced metabolic dysfunction. Moreover, *Aerococcus* was relatively enriched in the RHO group. This genus has been documented as an opportunistic pathogen in immunocompromised individuals [47], suggesting that excessive RPO intake may induce a microbial response distinct from that of moderate supplementation.

The correlation analysis further supports the potential interaction between gut microbiota alterations and host metabolic status. Although most associations did not remain statistically significant after BH-FDR correction, the overall trends suggest that microbial remodeling induced by RPO may be linked to changes in oxidative stress and lipid metabolism. Collectively, these findings support the possibility that moderate RPO supplementation may improve metabolic homeostasis partially through modulation of gut microbial composition and reduction of oxidative stress. Nevertheless, the present study was primarily based on 16S rRNA taxonomic analysis, and further metagenomic, metabolomic, and mechanistic studies are still required to clarify the causal relationships underlying these host–microbiota interactions.

In conclusion, RPO supplementation was associated with improved lipid metabolism, enhanced hepatic antioxidant capacity, and reduced liver injury in HFD-fed mice. Exploratory analyses suggest that low-dose RPO may exert more pronounced metabolic benefits than high-dose RPO, potentially due to the complex interplay of bioactive compounds and saturated fatty acids in RPO. Additionally, RPO supplementation was associated with dose-dependent alterations in gut microbiota composition; however, further functional studies are required to determine whether these microbial changes contribute causally to the observed metabolic improvements.

Certain limitations of the present study should be noted. First, the gut microbiota analyses were based on a relatively small cohort, which may have reduced the sensitivity for detecting subtle microbial differences, particularly after multiple testing correction in the correlation analyses. Second, this study evaluated RPO in isolation without considering its potential interactions with other dietary components, such as fiber or polyphenols, which may limit the applicability of the findings to real-world dietary contexts. Third, the present study relied primarily on 16S rRNA sequencing, which provides compositional rather than functional information; microbial metabolites such as SCFAs were not directly measured, and functional analyses such as metagenomics and metabolomics were not performed. Fourth, the observed microbiota–host associations were correlational in nature and should not be interpreted as causal relationships. Fifth, the 10-week experimental duration may not reflect long-term cardiometabolic outcomes, and longer-term studies incorporating additional cardiovascular biomarkers are warranted. Finally, this study was conducted in a mouse model, and the translatability of these findings to human populations remains to be established. Future studies should incorporate larger cohorts, direct comparisons with other dietary oils, multi-omics approaches, targeted metabolite analyses, and long-term human randomized controlled trials that account for the high inter-individual variability of the human gut microbiota to further elucidate the mechanisms underlying the metabolic effects of RPO and evaluate their clinical relevance.

## 5 Conclusions

In summary, this study demonstrates that RPO supplementation, particularly at a lower dose, attenuates HFD-induced metabolic disturbances in mice, as evidenced by improvements in serum lipid profiles, hepatic antioxidant capacity, and liver histology, along with partial restoration of gut microbiota composition. The observed differences between the RLO and RHO groups suggest a non-linear dose–response relationship, with the lower dose exerting more pronounced beneficial effects on several parameters. While these findings suggest a potential role of the gut–liver axis in mediating the effects of RPO, several limitations should be considered, including the relatively small sample size, the use of a mouse model, and the need for further mechanistic and clinical validation.
